# The free bilamellar autograft (FBA) procedure: A comprehensive case series of an alternative surgical approach to reconstruction of large eyelid defects

**DOI:** 10.3389/fsurg.2023.1038952

**Published:** 2023-02-24

**Authors:** Heather M. McDonald, Katherine A. McDonald, Hector McDonald

**Affiliations:** ^1^ Department of Ophthalmology, Western University, London, ON, Canada; ^2^ Division of Dermatology, University of Toronto, Toronto, ON, Canada; ^3^ Department of Ophthalmology, Eyelids Ottawa Surgical Centre, Ottawa, ON, Canada; ^4^ Department of Ophthalmology, University of Ottawa, Ottawa, ON, Canada

**Keywords:** full thickness eyelid defect, large eyelid defect, eyelid surgery, eyelid reconstruction, free bilamellar autograft, eyelid graft

## Abstract

**Purpose:**

The free bilamellar autograft (FBA) procedure involves harvesting a free, full-thickness section of eyelid tissue from one of the patient’s healthy eyelids to reconstruct a large defect of the involved eyelid. No vascular augmentation is employed. The purpose of this study was to determine the structural and cosmetic results of this procedure.

**Methods:**

A case series was performed, looking at patients who underwent the FBA procedure for large, full-thickness eyelid defects (>50% eyelid length) between 2009 and 2020 at a single oculoplastic surgical centre. Basal cell carcinomas most frequently met criteria for the procedure. OHSN-REB waived ethics approval. All surgeries were performed by one surgeon. A single operation, with surgical steps reported in detail, was completed with follow-up documentation at ideally 1 day, 1 week, 1 month, 3 months, 6 months, and 1 year. The mean follow-up period was 28 months.

**Results:**

Thirty-one patients (17 males, 14 females, mean age 78-years-old) were included in the case series. Comorbidities included diabetes and smoking. Most patients had known basal cell carcinomas removed from the upper or lower eyelid. The mean widths of the recipient and donor sites were 18.8 and 11.5 mm, respectively. All 31 FBA surgeries resulted in structurally functional, cosmetically pleasing, and viable eyelids. Six patients developed minor graft dehiscence, 3 developed an ectropion, and 1 developed mild superficial graft necrosis secondary to frostbite, which fully recovered. Three healing phases were identified.

**Conclusion:**

This case series adds to the currently sparse data on the free bilamellar autograft procedure. The surgical technique is clearly articulated and illustrated. The FBA procedure is a simple and efficient alternative to current surgical techniques in the reconstruction of full-thickness upper and lower eyelid defects. The FBA provides functional and cosmetic success, despite the absence of an intact blood supply, with decreased operative time and faster recovery.

## Introduction

The eyelid plays an essential role in protecting the globe, maintaining a stable tear film, and nourishing the cornea. It is a complex tissue composed of an inner layer of mucosa, semi-rigid connective tissue of the tarsal plate, muscle, blood vessels, nerves, secretory glands, and an outer layer of skin. The eyelid is considered a bilamellar structure with the orbicularis oculi and skin comprising the anterior lamella and the tarsal plate and conjunctiva comprising the posterior lamella (1). Large defects (>50% horizontal length) of the eyelid can arise from multiple conditions, most often post-excision of an eyelid skin cancer (2). Basal cell carcinoma (BCC) is the most common type of skin cancer, with 20% appearing in the periocular region (3). Reconstruction is required to recreate the original anatomy of the lid. Current standards for reconstructing large, full-thickness eyelid defects include a wide variety of surgical options (4, 5). Procedures include direct closure for smaller defects (<33%) and lateral cantholysis with Tenzel semicircular flaps for moderate-sized defects (33%–66%). However, when the defect is larger than 66%, a combination of an anterior graft with posterior flap, anterior flap with posterior graft, or anterior and posterior flaps have historically been required.

Traditional teaching states that only one lamella can be formed using a free graft, and the other must be a vascularized flap to minimize the risk of central necrosis (6, 7). Larger lid defects often require multi-step, time-consuming reconstructive procedures such as the Hughes tarsoconjunctival flap and the Cutler-Beard procedure. These surgeries require 2–8 weeks between steps, resulting in prolonged discomfort to the patient, temporary obstruction to their vision, a cosmetically displeasing appearance during this time, as well as increased operating room hours. These measures are a consequence of previous studies reporting high failure rates for full-thickness eyelid grafts without an augmented vascular supply (8). However, such a procedure represents the best possible option for reconstructing anatomy and appearance.

Composite grafts have been used by some surgeons. These are full-thickness eyelid grafts which are often manipulated or combined with a vascular bed. The anterior and posterior lamellae may be offset (9), a skin flap may be utilized in combination with an eyelid margin/tarsus/conjunctiva graft (10), the orbicularis oculi muscle may be removed (11), or a “sandwich” technique may be employed (the orbicularis functions as an advancement flap and is sandwiched between a free posterior and anterior lamellar graft) (12). The next step is exploring the use of a free, full-thickness eyelid graft. The periocular region is known to be well-vascularized, allowing for superior wound healing in the absence of supplemental vascular supply (13). In 2021, Tenland et al. published a small case series of 10 patients who underwent successful full-thickness eyelid grafting, a procedure rarely described in textbooks and journals (14–17). Although done independently, we enhance the data provided in the Tenland et al. case series (16) by presenting a larger case series of a similar procedure, including a broader range of comorbidities, phases of healing, and an emphasis on the surgical technique. As per Tenland et al., we refer to this as a free bilamellar autograft (FBA) procedure.

## Materials and methods

The single-center case series data was collected from patients operated on between February 2009 to September 2020 in Ottawa, Canada. All surgeries took place at one oculoplastic surgical centre and were performed by one surgeon (HM). All patient data and outcomes were documented in the clinic’s electronic medical record (THINK EMR). The Ottawa Health Science Network Research Ethics Board (OHSN-REB) waived ethics approval for the case series. The case series adhered to the tenets of the Declaration of Helsinki. All referred patients with large, full-thickness eyelid defects (>50% eyelid length) considered too large for direct closure were offered the FBA procedure. Patients with clinically suggestive BCC limited to the eyelid were preferentially selected over squamous cell carcinoma (SCC) because SCC behaves more aggressively in the periocular region. Furthermore, the cases of SCC that presented to the clinic often extended beyond the eyelid skin and therefore these patients were better candidates for alternative reconstructive procedures. Informed verbal and written consent for the procedure included: (1) a description of the procedure, (2) risks and benefits of the procedure, (3) alternative surgical options, and (4) an explanation that a second procedure using a standard eyelid reconstructive technique might be required to vascularize the donor tissue if the graft became compromised. All patients who were offered the procedure consented to proceed. No cases were withheld from data presentation. Both upper and lower eyelids with malignant tumors qualified for the FBA procedure, and any uninvolved eyelid could be used for the full-thickness donor tissue. The graft was harvested from either the ipsilateral or contralateral side, but from the uninvolved upper or lower eyelid. Written consent was obtained from those patients whose photos appear in this study.

### Procedure set-up

**Figure 1:**
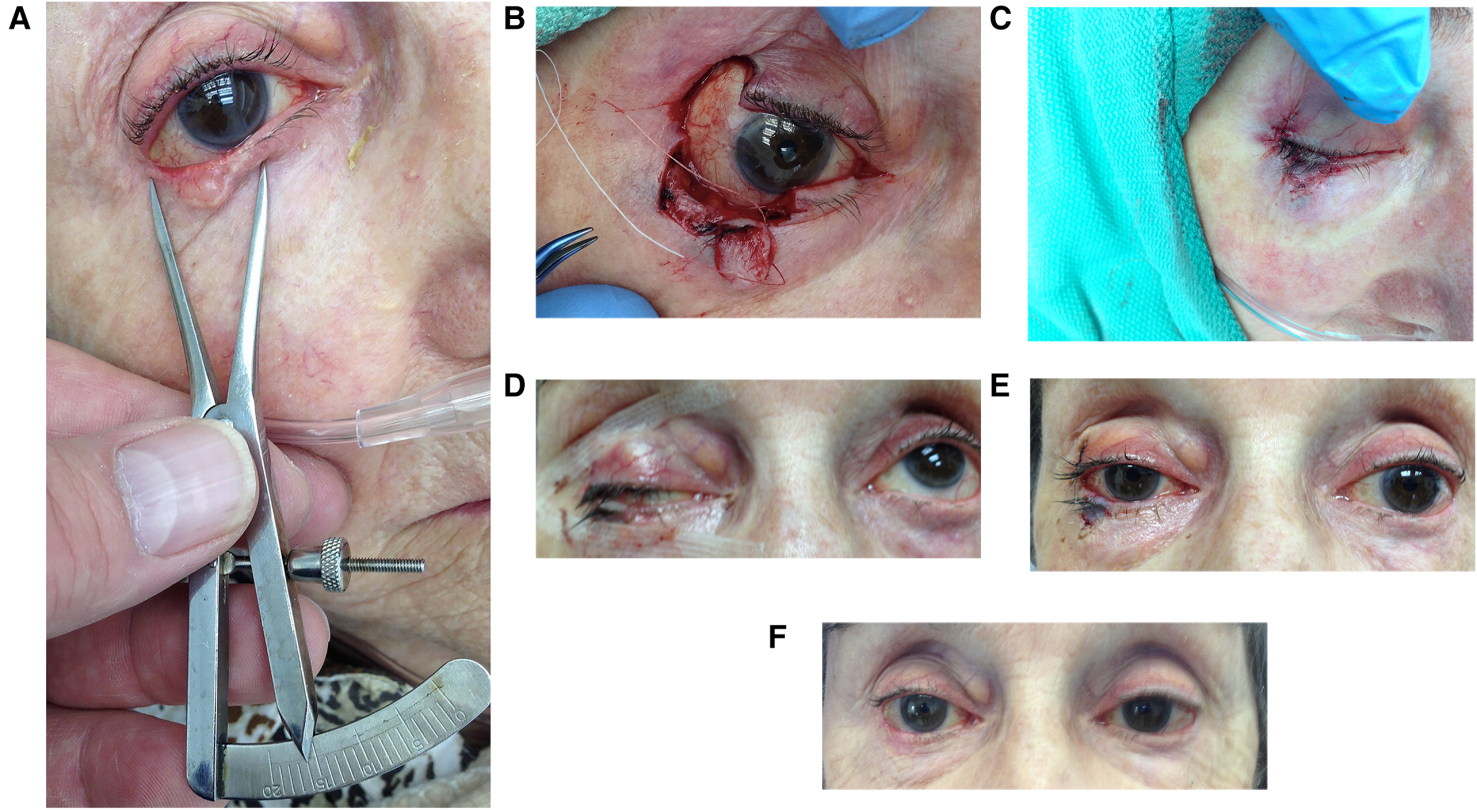
A stepwise photo series of the FBA procedure. (**A**) Preliminary measurement of tumor, (**B**) donor lid harvested and tumor resected with 4 mm margins, (**C**) immediate post-procedure repair of graft and donor site, (**D**) 24 h post-procedure with marked ptosis, (**E**) 6 days after surgery, (**F**) 3 months post-procedure with resolved ptosis.

Anesthesiologists used neuroleptic sedation for each patient with a combination of ketamine, midazolam, fentanyl and propofol. The surgeon used loupes with a 3.3X magnification and a headlight. The tumour was assessed, measured (Figure 1A), and marked with standard four millimetre surgical margins for BCC and seven millimeter surgical margins for melanoma *in situ* (MIS). The width of the excised area was documented. The donor tissue width was estimated and marked (Figure 1B). The donor lid was then stretched horizontally, ensuring that the secondary defect could undergo direct closure. One drop of topical anesthesia was placed in each eye and the operative site was prepared with controlled use of chlorhexidine to limit the risk of corneal toxicity. The surgeon performed subcutaneous infiltration of the tumor and donor sites using lidocaine 2% with epinephrine 1:100,000; 2 ml or less per eyelid. All tissue was handled with 0.5 mm toothed forceps to preserve its architecture and integrity.

### Procedure

**Figure 2:**
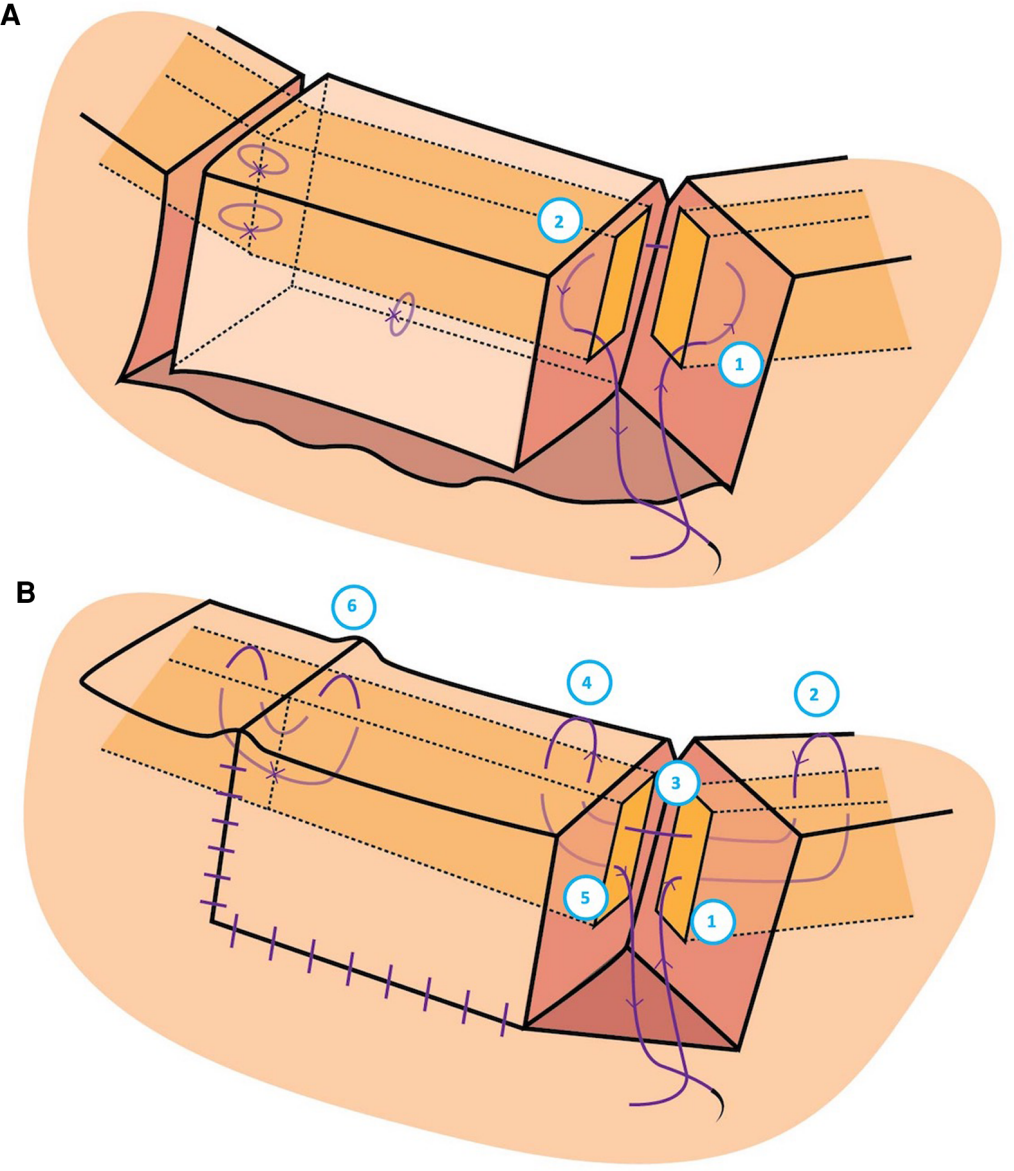
Graphic illustration of the suturing technique used to secure the donor eyelid to the host eyelid. (**A**) The needle was passed: (1) at the surgical incision of the host just anterior to the tarsus but posterior to skin, through the host tarsus, and exiting just posterior to the tarsus but anterior to the palpebral conjunctiva to create a buried suture, then (2) at the surgical incision of the donor just anterior to the palpebral conjunctiva and posterior to the tarsus, through its tarsal plate and exiting just posterior to the eyelid skin. (**B)** The needle was passed: (1) Through the vertical edge of the host tarsus about 4 mm vertically from the eyelid margin, through the tarsus towards the palpebral fissure, aiming for the grey line of the host margin about 3 mm horizontally from the vertical incision; (2) Through the host grey line aiming back into the host tarsus; (3) Out of the host tarsus and into the donor tarsus about 2 mm vertically from the eyelid margin; (4) Back through the donor grey line of the eyelid margin; and (5) Passed back into the tarsus, coming out at the same level it went in to the host lid; (6) Creating a raised host-donor interface.

Tumour excision and reconstruction occurred consecutively on the same day with post-operative pathology assessment. Each tumor was excised using Stevens scissors at the pre-delineated vertical and horizontal margins with clean single incisions through the full-thickness of the eyelid (Figure 1B). The autograft was always taken from the lateral aspect of the lid. Gentle monopolar cautery was carried out. Instruments were not replaced prior to reconstruction. Stevens scissors were used to create full-thickness single, deliberate, straight medial and lateral vertical incisions, joined by a clean horizontal cut to form a rectangular bilamellar composite graft (Figure 1B). The graft was immediately anchored in the recipient site with one 6–0 polyglactin suture (on a P1 needle) through both the donor and host eyelid skin and tarsal plates. 6–0 polyglactin sutures were placed at the recipient site near the eyelid margin of the two vertical interfaces and centrally at the horizontal interface, ensuring proper alignment of the donor and host lid margins under minimal tension. The two vertical lid margins were then approximated with 7–0 polyglactin (on a P1 needle) (Figure 2B), creating a raised interface. The procedure was repeated for the other vertical donor-host interface. The skin was gently approximated with simple interrupted 7–0 polyglactin sutures. The posterior lamella was not manipulated as proper alignment was achieved with the 6–0 polyglactin sutures. Repair of the donor site was performed with a 6–0 polyglactin suture attaching the tarsus to the periosteum of the lateral orbital rim. Skin was closed using 7–0 Polyglactin interrupted sutures (Figure 1C).

### Postoperative care

Tobramycin/dexamethasone ointment was applied to all suture lines four times a day for the first week and then at bedtime for the second week after surgery. A patch was applied for the first 24 h and a shield was worn at bedtime for the first month following surgery. Patients were followed closely, ideally with appointments at 1 day (Figure 1D with acute ptosis), 1 week (Figure 1E), 1 month, 3 months (Figure 1F), 6 months, and 1 year after the operation. The mean follow-up period was 28 months for this case series (Table 1), and some patients were seen beyond the data collection period with no concerns. Non-identifying pictures were taken of the eyelid at each follow-up to assess for structural integrity, appearance, and viability of the autograft. Phases of healing were documented. Photos were subsequently assessed by all authors.

**Table 1:** Free bilamellar autograft measurements, locations, and complications, listed according to increasing size of recipient site.

Patient #	Width of pre-excision recipient site (mm)	Width of pre-excision donor lid (mm)	Donor to recipient (±additional technique)	Length of follow up (months)	Complications
24	15	10	RUL to RLL, S	11	-
2	16	12	LUL to LLL	3	-
6	16	12	LUL to LLL	103	-
7	16	12	RUL to RLL	17	-
10	16	12	LUL to LLL	3	-
17	16	12	LLL to RLL	59	-
18	16	12	LUL to LLL	67	BCC recurrence at 2 and 6 years, re-excised.
3	17	13	LUL to LLL, C	55	-
5	17	13	RUL to RLL	21	-
8	17	13	LUL to LLL	11	-
9	17	12	LUL to LLL	7*	-
12	17	12	LUL to LLL	12	Minor traumatic graft dehiscence, repaired
16	17	10	RUL to RLL	3	-
20	17	10	RUL to LLL	5	Minor traumatic graft dehiscence, repaired
1	18	13	RUL to RLL	16	-
19	18	12	RUL to LUL	34	-
23	18	10	RUL to RLL	36	Chalazion
31	18	10	LUL to LLL	14	Ectropion, repaired
4	18.5	12	LUL to LLL	99	Distichiasis A new primary BCC lateral to previous BCC at 6 years
11	19	12	LUL to LLL, C	13	-
14	19	11	RUL to RLL, C	20	-
15	19	9	LUL to LLL	6	Chalazion
22	20	10	RUL to RLL	4	Cyst inferior to autograft
25	20	12	RUL to RLL	34	Cyst in autograft
27	20	10	RUL to RLL, F	6	Traumatic donor site dehiscence, no repair required
13	22	13	RUL to RLL, S	24	-
26	22	12	LUL to LLL	74	Superficial necrosis secondary to frostbite Ectropion. Mild corneal exposure, required tarsorrhaphy
30	24	12	LUL to LLL	13	Minor graft dehiscence, repaired
21	25	11	LUL to LLL	58	Mild ectropion, no repair required
29	25	11	RUL to RLL, F	29	Minor graft dehiscence, repaired
28	27	11	RUL to RLL, F	13	Minor graft dehiscence, repaired
Mean (±SD)	18.8 (±3.0)	11.5 (±1.1)		28 (±28)	

Surgeries performed from 2009 to 2020. Note that the height of all autografts was approximately 8 mm. RUL, right upper lid; LUL, left upper lid; RLL, right lower lid; LLL, left lower lid; C, cantholysis; F, mini rotational temporal flap; S, canalicular stent (C, F, and S were performed on the recipient eyelid).

* Referred to dermatology and continues to be followed.

### Pathology

The excised tissue was sent to pathology for analysis after surgery with standard vertical paraffin sections.

## Results

Thirty-one patients were included in the case series: 14 females (14/31; 45%) and 17 males (17/31; 55%), with a mean age of 78-years-old (SD ± 9 years) (Table 2). Patients had a variety of comorbidities and 13 were on blood thinners, as indicated in Table 2. The average width of the tumor excision (recipient site pre-excision) was 18.8 mm (SD ± 3.0), ranging from 15 to 27 mm. The average donor site width pre-excision was 11.5 mm (SD ± 1.1 mm), ranging from 9 to 13 mm, and approximately 8–10 mm in height (Table 1). For very large recipient sites in 3 cases, a mini temporal rotation flap was also performed to help fill in the defect. A cantholysis was performed at the recipient site when the lid was under too much tension in an additional 3 cases. Two patients required a canalicular stent based on the location of their tumors. The upper eyelid was used for the ipsilateral lower lid most commonly, but also grafted to the contralateral lower or upper lid. The lower eyelid was grafted to the contralateral lower lid for one patient.

**Table 2:** Patient demographics, diagnosis, past medical history, and anticoagulants.

Patient #	Age at surgery	Sex	Diagnosis	Medical history	Blood thinners
1	85	F	BCC	CHF, arthritis	ASA
2	87	M	BCC	HTN	ASA
3	81	F	BCC	CAD, HTN, asthma, arthritis	ASA, Clopidogrel
4	79	F	BCC	HTN, previous eyelid surgery	None
5	85	F	BCC	CAD, hypothyroid, HTN, laser eye surgery, arthritis, HSV, previous eyelid surgery	ASA
6	67	F	BCC	HTN, myotonic dystrophy	ASA
7	79	F	BCC	HTN, diverticulitis, arthritis, asthma/COPD, smoker	None
8	87	M	BCC	GPA, COPD, previous eyelid surgery, smoker	None
9	58	M	MIS	DM	None
10	83	M	BCC	CAD, HTN, DM, asthma/COPD	None
11	82	F	BCC	HTN, Parkinson’s	None
12	61	M	BCC	Glaucoma	ASA
13	79	F	BCC	Glaucoma, previous eyelid surgery	None
14	88	F	BCC	CVA, previous eyelid surgery	None
15	79	M	BCC	DM, HTN, previous eyelid surgery, laser eye surgery, retinal detachment, smoker	ASA
16	85	F	BCC	Glaucoma	ASA
17	78	M	BCC	Previous eyelid surgery	None
18	58	M	BCC	Charcot-Marie Tooth, cancer, arthritis, previous eyelid surgery	None
19	84	F	BCC	HTN	None
20	87	F	BCC	HTN, arthritis, DM, HSV, monocular secondary to VZV	ASA
21	78	M	BCC	CAD, HTN, arthritis, thyroid disease, asthma/COPD	None
22	92	M	BCC	Arthritis	None
23	78	M	BCC	Cancer, HSV, hepatitis	None
24	79	F	BCC	CAD, thyroid disease, HSV	None
25	79	F	BCC	HTN, kidney disease, arthritis, thyroid disease, previous retinal detachment, HSV	ASA
26	66	M	BCC	Skin cancer, COPD, previous eyelid surgery, smoker, alcohol-use disorder	Clopidogrel, ASA
27	75	M	BCC	CAD, stroke, GERD, kidney stones, smoker	ASA
28	60	M	BCC	CAD, smoker, marijuana use	None
29	87	M	BCC	Asthma, DM, hepatitis, arthritis	None
30	66	M	BCC	HTN, COPD, CAD, arthritis, smoker, marijuana use	None
31	73	M	BCC	HTN, DM	Eliquis, ASA
Mean (±SD)	78 (±9)				

Patients listed in chronological order of surgery. BCC, basal cell carcinoma; MIS, melanoma in situ; CHF, congestive heart failure; HTN, hypertension; CAD, coronary artery disease; GPA, granulomatosis with polyangiitis; COPD, chronic obstructive pulmonary disease; DM, diabetes mellitus; CVA, cerebral vascular accident; HSV, herpes simplex virus (cold sores); ASA, aspirin.

In the surgical center’s region, it is not protocol for dermatopathologists to provide histopathological margin measurements for BCC. However, the reports stated that the peripheral and deep margins were negative for all patients. The case with MIS had 5 mm margins on histopathology. Radical excision was achieved in all cases.

### FBA eyelid function and complications

The FBA procedure results demonstrated success with respect to structural integrity, appearance, and viability of the full-thickness composite graft. No complications occurred during the procedure. The sutures did not have to be removed and never caused friction between the lid and cornea. All patients were satisfied with the functional and cosmetic result of their surgery. In general, complications were more common in patients who had a recipient site larger than 17 mm (Table 1).

A chalazion developed in the autograft of two eyelids post-surgery, demonstrating that its Meibomian glands likely remained functional. Three patients developed mild ectropion, two of which required repair. There was initial ptosis in many patients due to shortening of the donor eyelid’s horizontal length; however, this resolved over weeks. Lashes were generally lost or reduced in number on the grafted eyelid. Two patients developed cysts within or adjacent to the autograft that were inconsequential. Six patients developed minor dehiscence of the autograft, three of which were traumatic in nature due to rubbing the eye, wearing swimming goggles, and using a shield with a string post-operatively. Approximation sutures were required in five of these patients.

One patient had a recurrence of BCC at the recipient site many years after excision (Table 1). A different patient had a second primary BCC develop lateral to the excision site. One patient experienced superficial necrosis in the middle inferior aspect of the graft, after developing frostbite of the face. The graft ultimately healed but led to a mild ectropion. The patient required a temporary tarsorrhaphy due to exposure keratitis. No patients developed postoperative entropion, eyelid notching, distichiasis, trichiasis, lanugo hair irritation, donor lid contraction, dry eyes, or other complications (Table 1).

### Phases of autograft healing and cosmesis

**Figure 3:**
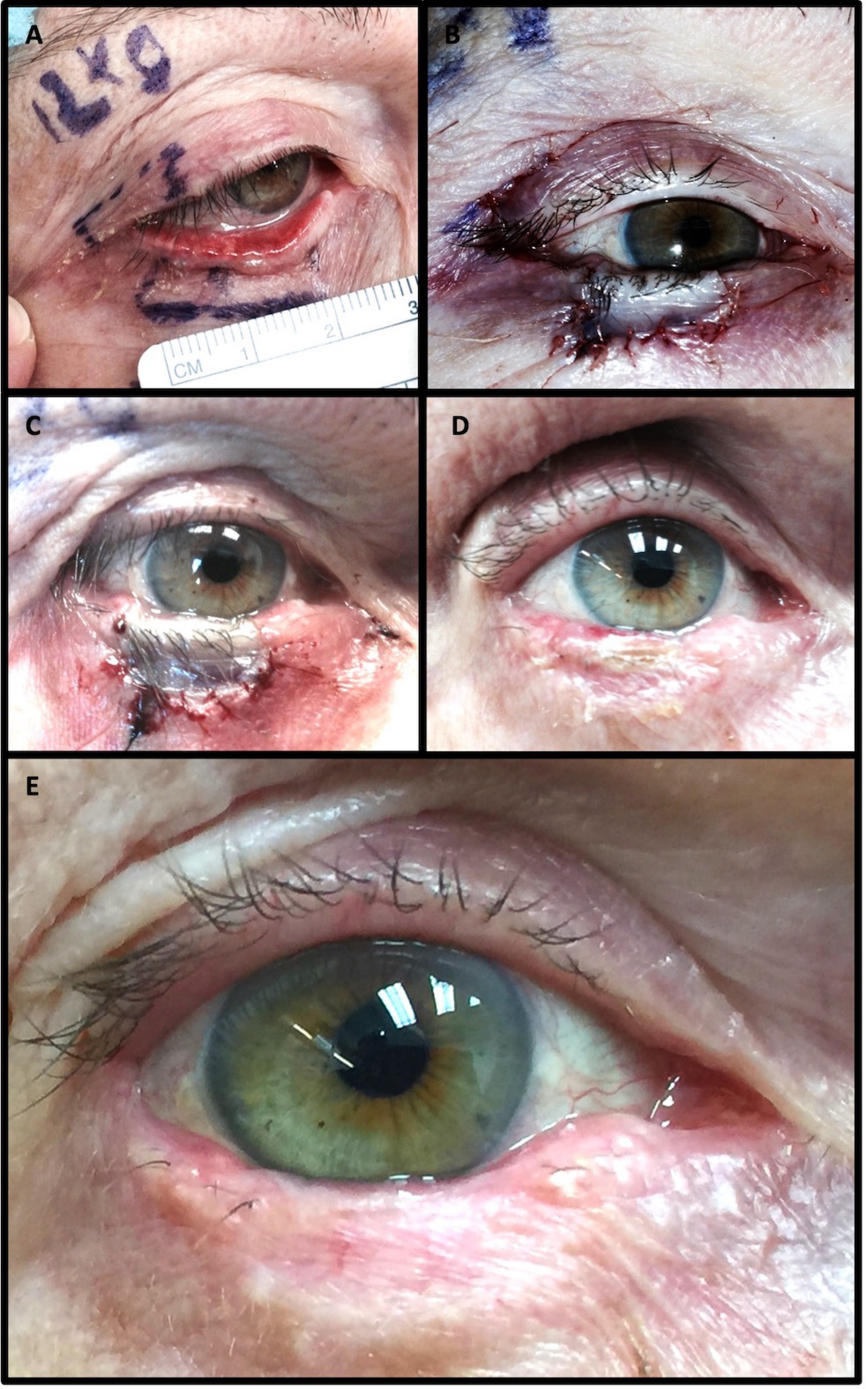
Healing phases of right-upper to right-lower FBA. (**A**) Pre-operative markings. (**B**) White phase immediately post-FBA. (**C**) Blue phase 4 days post-FBA. (**D**) Pink phase 1.5 months post-FBA. (**E**) Final outcome 6 months post-FBA.

The FBA procedure heals in three observable phases: the white phase, the blue phase, and the pink phase (Figure 3). Using the pictures taken at each follow up appointment and patient feedback, it was approximated that the white phase lasted from the time of operation to between 24 and 48 h post-operation, the blue phase lasted from 24 to 48 h and 7–12 days post-surgery, and the pink stage started around day 7–12 post-surgery with visible blood vessels in the autograft. The pink colour slowly faded over weeks to months.

## Discussion

The FBA procedure offers an alternative method for surgeons to include in their armamentarium for treating large, full-thickness eyelid defects. Although previously called a free bilamellar autograft, it might be considered an autologous transplant. The term transplant could be favoured over graft due to the transfer of a full-thickness, unaltered piece of an accessory ocular structure, including skin, muscle, tarsal plate, secretory glands, and mucosal tissue, from one location to another with no enhanced vascular supply.

To the best of our knowledge, there is only one previous case report and one previous case series describing full-thickness free bilamellar autografts that do not require any augmented vascular supply or pedicle (16, 17). Memarzadeh et al. described a single case in which a successful full-thickness free graft was transferred from the ipsilateral lower eyelid to the upper eyelid defect (17). Tenland et al. reported a small series of 10 patients who underwent a similar procedure, but always used the contralateral eyelid as the donor (16). Although surgical technique is not the emphasis in Tenland et al.’s series, there are many similarities and results are comparable (16). Tenland et al. reported success in all ten of their cases (16). One of their cases (10%) developed dehiscence due to excess tension. Three of our cases (10%) experienced mild traumatic dehiscence, and three (10%) developed minimal spontaneous dehiscence. Two of Tenland’s patients (20%) had mild ectropion not requiring repair, and three patients (10%) in our series developed the same, two of which required repair. Three of Tenland’s recipient sites (30%) required a rotational flap to reduce tension; another 5 (50%) donor sites required the same. Only 3 of our patients (10%) required a rotational flap, but an additional 3 (10%) required cantholysis to reduce tension. In all cases under tension in our series, the donor and recipient sites were ipsilateral. Tenland used a pentagonal excisional approach, whereas all our grafts were rectangular. The authors of both the previously published case report and series recommended that a larger series would be beneficial in supporting this procedure (16, 17). This case series was started prior to the Tenland case series and conducted independently, resulting in the subtle differences in surgical technique (16).

Current standards for reconstruction are constrained by a necessitated vascular supply, often requiring multiple surgeries (6). This is of particular concern in monocular patients, or pediatric patients who could develop amblyopia. The FBA is a single-step procedure that minimizes operating room time and post-procedural patient discomfort. Functional outcomes of currently used procedures can be hampered by complications including madarosis, ptosis, superior eyelid entropion, irregularity of the lid margin, inferior eyelid cicatrisation and retraction, as well as necrosis of the flap (6, 18). Madarosis was common in the FBA procedure, ectropion infrequently occurred, and one patient developed superficial necrosis secondary to frostbite. Although shortening of the horizontal lid length caused ptosis of the donor lid was observed initially, this resolved over a few weeks in all cases (19).

The Meibomian gland function is often lost with classic eyelid reconstruction techniques. This is known to contribute to ocular surface disease (20). Two patients in this case series developed a chalazion within their grafted tissue, suggesting that Meibomian gland function may be preserved following the FBA procedure, theoretically resulting in a more stable tear film (21). This is further supported by no patients developing dry eyes. One patient had recurrence of their BCC years later, resulting in a recurrence rate of 3.2%. This is consistent with the known recurrence rate of 2%–5% in radical resections (22). The periocular area is an area of higher risk for BCC recurrence (23). Therefore, it may also be compared to the 2.9% recurrence rate in a 2020 meta-analysis reviewing recurrences rates of periocular BCC following excision with Mohs micrographic surgery (24). Although iatrogenic implantation has been raised as a potential concern if instruments are not replaced after the extirpative phase of a cancer surgery, there is no literature to suggest that instruments used during margin-negative resection on BCC can cause tumour seeding or recurrence (25). Furthermore, the primary and secondary defects are often in proximity for this procedure, rendering complete separation impossible.

Patients who underwent the FBA procedure had a range of comorbidities including diabetes and tobacco use, which can be associated with poor wound healing and flap necrosis. (26)5. Smoking cessation prior to the procedure would be ideal. However, the presence of such comorbidities affecting microcirculation in patients with satisfactory FBA results supports that the procedure is robust.

The overall success of the FBA procedure suggests that an enhanced vascular supply is not always necessary when an intact, unaltered, bilamellar, full-thickness eyelid donor is auto-grafted. The eyelid’s naturally rich vascular supply provides the potential for plasmatic imbibition, inosculation, and revascularization of the arterial arcade. The procedure allows for the survival of all aspects of the organ except for the cilia.

### Phases of healing

The autograft tissue followed the same healing processes as previously described for skin transplants and composite grafts. The white phase demonstrates ischemia induced by surgery and injected epinephrine. Plasmatic imbibition ensues, wherein the FBA absorbs nutrients and oxygen from the host interface (27, 28). The blue phase reveals the beginning of inosculation with an arterial-venous mismatch leading to mild venous congestion and a dark blue-purple discolouration. The pink phase demonstrates angiogenesis secondary to the eyelid’s rich vascular supply, with reliable arterial perfusion and venous drainage (Figure 2) (29). In the final stages of healing there is epidermal proliferation.

These phases of healing may be further supported by the underlying tear film, which provides lubrication, antimicrobial support, promotes wound healing, suppresses inflammation, and scavenges free radicals (30). The aqueous-mucin tear layer contains growth and supportive factors, some of which participate in epithelial or stromal wound healing as well as angiogenesis (e.g., vascular endothelial growth factor promoting blood vessel formation from pre-existing vessels) and neovascularization (e.g., epidermal growth factor; EGF; promoting *de novo* formation of blood vessels in addition to connection from pre-existing) (31). Platelet-derived growth factors and transforming growth factor-beta potentiate tissue repair *in vivo* and EGF is a known promotor of adipose graft survival as a potent stimulator of neovascularization (32, 33).

Cold outdoor temperatures and physical trauma (e.g., swimming goggles) in the early phases of healing caused mild dehiscence and early signs of superficial necrosis, respectively (Table 1). There is likely an upper limit to the width of recipient site being repaired, which could not be determined in this study. While ipsilateral eyelids were used in this study as donor tissue, it may be wise to preserve this tissue and use contralateral lids in the event that a bridging flap is required later on. A potential limitation that was not encountered in this study is irradiated tissue. If radiation is being used in the eyelid region, the FBA procedure should not be considered, as it could be detrimental to flap survival (34).

The pathology was assessed with standard vertical paraffin sections, in which only 1%–2% of the margin can be evaluated by a pathologist (24). Intraoperative horizontal (*en face*) frozen sections would allow for complete margin assessment (24), with the potential to further reduce recurrence rates.

This study is limited by the fact that it is a retrospective case series without a comparison group. There was no specific grading scale used to document the outcomes for each case; however, they were all assessed by one surgeon for consistency and photos were assessed by all authors to improve objectivity. Unfortunately, the height of the recipient and donor sites were not consistently measured and therefore cannot be specifically addressed, but were approximately all in the range of 8–10 mm. Finally, the number of patients in the case series is reasonable and a notable contribution to the current literature, but still too small to draw definitive conclusions regarding rates of complications.

## Conclusion

The FBA procedure was introduced in 2021 by Tenland et al. as a simple and efficient method of reconstructing large, full-thickness eyelid defects (16). This case series adds to the literature and demonstrates that the procedure is robust and patients with a wide range of comorbidities are eligible. The ultimate structural and cosmetic results were almost universally successful across the 31 patients in this case series. Consequently, the FBA procedure may be considered as a reconstructive technique for large full-thickness eyelid defects in select patients.

## Data Availability

The original contributions presented in the study are included in the article/Supplementary Material, further inquiries can be directed to the corresponding author.
